# LncRNA PGM5-AS1 inhibits non-small cell lung cancer progression by targeting miRNA-423-5p/SLIT2 axis

**DOI:** 10.1186/s12935-024-03402-5

**Published:** 2024-06-20

**Authors:** Jiajun Wang, Jun Ye, Yuxue Dang, Shun Xu

**Affiliations:** 1 Department of Thoracic Surgery, The First Hospital of China Medical University, No. 155, North Nanjing Street, Shenyang, 110001 Liaoning China; 2 Department of Radiology, Shengjing Hospital of China Medical University, No. 36, Sanhao Street, Shenyang, 110004 China

## Abstract

Non-small cell lung cancer (NSCLC) is a common and aggressive primary malignancy worldwide. Dysregulation of long non-coding RNAs (lncRNAs) has been shown to play an essential regulatory role in multiple cancers. However, the role of PGM5-AS1 in NSCLC remains unclear. Here, we found that PGM5-AS1 was down-regulated in NSCLC tissues and cells. Furthermore, reduced PGM5-AS1 expression levels were associated with larger tumor size, positive lymph node metastasis, advanced TNM stage and worse prognosis. We found that overexpression of PGM5-AS1 inhibited cell proliferation and metastasis, and induced apoptosis and G0/G1 cell cycle arrest in NSCLC cell lines. Using dual luciferase gene reporter and RNA immunoprecipitation assays, we confirmed that miR-423-5p interacted with PGM5-AS1, and that their expression levels were negatively correlated in NSCLC tissues. miR-423-5p was also found to reverse PGM5-AS1-induced malignant biological behavior. Moreover, we identified slit guidance ligand 2 (SLIT2) as a target gene of miR-423-5p. Using a dual luciferase gene reporter assay, we confirmed the regulatory relationship between SLIT2 and miR-423-5p and demonstrated that their expression levels were negatively correlated. Our rescue experiments showed that SLIT2 knockdown reversed miR-423-5p-mediated effects. Overall, this study identifies PGM5-AS1 as a potential prognostic biomarker for NSCLC and shows that PGM5-AS1 suppresses NSCLC development by regulating the miR-423-5p/SLIT2 axis.

## Introduction

Lung cancer was the most commonly diagnosed cancer in both mortality and incidence worldwide in 2018 with 2,093,876 new cases and 1,761,007-associated deaths [1]. Non-small-cell lung cancer (NSCLC) is the main type of lung cancer, accounting for 85% of all patients [2]. Squamous cell carcinoma, adenocarcinoma and large cell carcinoma are three most common subtypes of NSCLC [3]. Most early-stage NSCLC patients can be cured with surgical resection [4]. However, the outcomes of patients diagnosed at an advanced stage or with tumor metastases are generally unsatisfactory due to the fact that surgical resection is not an option for these patients [5]. Many studies have aimed to elucidate the molecular mechanisms of NSCLC, and multiple tumor-related genes have been found to be involved in NSCLC progression. However, the precise molecular mechanisms of NSCLC remain unclear. Therefore, it is critical to identify novel biomarkers and therapeutic targets for improving the clinical outcome of patients with NSCLC.

Long non-coding RNAs (lncRNAs) are a class of non-coding RNAs that are longer than 200 nucleotides in length [6, 7]. In recent years, accumulating evidence has indicated that lncRNAs involved in regulating cell apoptosis, proliferation, invasion and drug resistance are often aberrantly expressed in various human cancers [8–10]. LncRNAs act by interacting with miRNAs, another evolutionarily conserved single stranded non-coding RNAs that are approximately 21–24 nucleotides in length, and thus function as competing endogenous RNAs (ceRNAs). By competitively binding to miRNAs, lncRNAs inhibit miRNA expression, thereby reducing the regulatory effects of miRNAs on their target genes and promoting mRNA expression [11, 12]. For example, MALAT1 promotes cell proliferation, migration, and invasion of NSCLC through sponging miR-503-5p [13]. Similarly, MLETA1 is a cancer-related lncRNA that promotes the malignant biological behavior of NSCLC by sponging miR-186-5p and miR-497-5p, resulting in increased EGFR and IGF1R expression respectively [14].

LncRNA PGM5-AS1 (NR_121191.1) is an antisense lncRNA that contains 706 nucleotides and is located at human chr9:68355164–68357866. Previous studies have reported that PGM5-AS1 is down-regulated in clear cell renal cell carcinoma (ccRCC), esophageal squamous cell carcinoma (ESCC) and colorectal cancer (CRC) [15–17]. Lower PGM5-AS1 expression levels in ccRCC and ESCC patients have been associated with a shorter survival time [15, 16]. Moreover, TP53 has been shown to induce the expression of PGM5-AS1, while PGM5-AS1 was found to inhibit ESCC proliferation and invasion through regulation of the miR-466/PTEN axis [16]. PGM5-AS1 has also been shown to regulate the proliferation and invasion of CRC cells by acting as a sponge for miR-100-5p and increasing SMAD4 expression levels [17]. However, the expression, function and molecular mechanisms of PGM5-AS1 in NSCLC have not been clarified until now.

Here, we show that PGM5-AS1 is significantly down-regulated in NSCLC tissues and cell lines, and that overexpression of PGM5-AS1 suppresses cell proliferation, migration and invasion in NSCLC. We also demonstrate that, by acting as a miRNA sponge, PGM5-AS1 binds to miR-423-5p to regulate expression of its target gene slit guidance ligand 2 (SLIT2), a tumor-suppressor in NSCLC and other types of cancer [18–20]. Our findings identify the PGM5-AS1/miR-423-5p/SLIT2 axis as a potential underlying mechanism of NSCLC progression and thus provide a potential target for the prevention or treatment of NSCLC.

## Materials and methods

### Sample collection and ethics statement

NSCLC tissues and matched normal adjacent tissues (NATs) were obtained from 50 patients who underwent radical resection of NSCLC in the Thoracic Surgery Department at the First Hospital of China Medical University (Shenyang, China) and had not received any treatment before surgery. All samples were histopathologically confirmed and frozen immediately in liquid nitrogen and stored at − 80 °C. The histological grade was staged according to the Tumor, Node, and Metastasis (TNM) staging Classification. This study was approved by the Research Ethics Committee of China Medical University (Shenyang, China) and was developed according to the principles of the Declaration of Helsinki. Tissue samples were harvested from 50 patients after obtaining their informed consent.

### Cell culture

H460, A549, H1975, H1299, HCC-827, SK-MES-1 and 16HBE cell lines were purchased from the Cell Culture Center of Chinese Academy of Medical Sciences (Beijing, China). The PC-9 cell line was purchased from KeyGen Biotech (Nanjing, China). SK-MES-1 cells were maintained in MEM (KeyGen Biotech), while the other cell lines were maintained in RPMI 1640 (KeyGen Biotech). All cell culture media were supplemented with 10% fetal bovine serum, 1% penicillin and streptomycin. Cells were cultured at 37 °C in an atmosphere of 5% CO_2_.

### Cell transfection

Cells in the logarithmic growth phase were used for transfection. Cells stably overexpressing PGM5-AS1 (LV-PGM5-AS1) and negative control cells (LV-NC) were generated using lentiviral vectors (GenePharma, Shanghai, China) and screened with puromycin. SLIT2 siRNA, miR-423-5p mimics, miR-423-5p inhibitor and their NC were all purchased from Synbio Tech (Suzhou, China). Transfections were carried out using Lipofectamine 3000 reagent (Invitrogen, Carlsbad, CA, USA) according to the manufacturer’s protocol.

### RNA isolation, reverse transcription, and quantitative real-time PCR (qRT-PCR)

Total RNA was isolated from tissues and cells using TRIzol reagent (Invitrogen Life Technologies, Carlsbad, USA) according to the manufacturer’s instructions. Total RNA was reverse transcribed into complementary DNA using the PrimeScript^™^ RT Reagent Kit with gDNA Eraser (TaKaRa, Dalian, China).

mRNA and miRNA expression levels were assessed by qRT-PCR using TB Green^®^ Premix Ex Taq^™^ (TaKaRa) in a final volume of 20 μL containing 10 μL 2 × TB Green Premix Ex Taq, 0.5 μL forward primers, 0.5 μL reverse primers (10 μM), 7 μL nuclease-free water, and 2 μL diluted reverse transcription products. The reaction was amplified using 1 cycle at 95 °C for 30 s, followed by 45 cycles at 95 °C for 5 s and 60 °C for 30 s.

The Bulge-Loop^™^ miRNA qRT-PCR Primer System (Ribobio, Guangzhou, China) was used to determine the expression levels of miR-423-5p, miR-4709-3p, miR-3065-5p, miR-4766-3p, miR-5701, miR-452-3p, miR-3688 and miR-587, while U6 acted as the internal control. The primer sequences for these miRNAs cannot be listed because they involve company patents.

The other primers used in our study were as follows: PGM5-AS1 forward primer: 5′-TGGTACTTTCAGCCTGTCCG-3′ and reverse primer: 5′- CAGACGGCTTCAGTGGTTGT-3′; SLIT2 forward primer: 5′-GAGAATTTGTCTGCAGTGGTCA-3′ and reverse primer: 5′-AGCTCCAGGAGGGATGACTT-3′; and GAPDH forward primer: 5′-CGGATTTGGTCGTATTGGG-3′ and reverse primer: 5′-CTGGAAGATGGTGATGGGATT-3′ (Sangon Biotech, Shanghai, China).

### Subcellular fractionation

Cell nuclear and cytosolic RNA were isolated using the Cytoplasmic and Nuclear RNA Purification Kit (Norgen Biotek, ON, Canada) according to the manufacturer’s instructions. PGM5-AS1, GAPDH and U6 expression levels in the cytoplasmic and nuclear fractions were determined by qRT-PCR.

### Fluorescence in situ hybridization (FISH)

The red FISH Probe of PGM5-AS1 (5′-AUAGUCCCUCUGCCCCGUGCCCU-3′) and FISH kit were purchased from Jijia Biotechnology (Shenyang, Liaoning, China). NSCLC and normal tissue specimens were subjected to FISH assay according to the manufacturer’s instructions.

### Cell counting kit-8 (CCK-8) proliferation assay

Cell proliferation was measured using the CCK-8 assay (Beyotime, Shanghai, China). Cells (3.5 × 10^3^ cells/well) were seeded into wells and cultured for 24, 48, 72 and 96 h. At each time point, 10 μL CCK-8 reagent was added to each well and incubated for 90 min at 37 °C. The absorbance was measured at a wavelength of 450 nm using a microplate reader.

### Colony formation assay

The colony-forming abilities of H460 and PC-9 cells were measured using the colony formation assay. Cells (600 cells/well) were cultured in 6-well plates for 14 days, then fixed with 4% paraformaldehyde and stained with crystal violet. The colonies in each well were photographed and counted using ImageJ software.

### 5-ethynyl-2′-deoxyuridine (EdU) assay

Cell proliferation was measured using the BeyoClick^™^ EdU-555 Kit (Beyotime) according to the manufacturer’s instructions. Images of the EdU-stained cells were captured by fluorescence microscopy and the number of EdU-positive cells were counted using ImageJ software.

### Flow cytometry

Cellular apoptosis and the cell cycle were assessed using the Annexin V-APC/PI Apoptosis Detection Kit (KeyGen Biotech) and Cell Cycle Detection Kit (KeyGen Biotech) according to the manufacturer’s instructions, and analyzed by flow cytometry.

### Transwell assay

The metastastic ability of the cells was evaluated using Transwell polycarbonate membranes (Corning, New York, USA). For the invasion assay, the upper Transwell chamber was coated with 50 μL diluted Matrigel (BD Bioscience, San Jose, CA) containing 5 μL Matrigel and 45 μL serum-free medium. For the migration assay, the upper chamber was not coated with Matrigel. Cells (5 × 10^4^ cells in 200 μL serum-free medium) were seeded into the upper chamber, while 600 μL medium containing 10% fetal bovine serum was placed in the lower chamber. Cells were incubated for 24 h at 37 °C, then the medium in the upper chamber was removed and cells were fixed with 100% methanol for 10 min. Samples were stained with hematoxylin for 3 min and eosin for 30 s. Non-invading cells were removed from the upper surface of the upper membrane with a cotton swab. The number of cells that had invaded through the membrane were counted in three randomly selected visual fields.

### Argonaute2 (AGO2) RNA immunoprecipitation (RIP) assay

The AGO2-RIP assay was performed using the MAGNA RIP^®^ Kit (Sigma-Aldrich, Darmstadt, Germany) according to the manufacturer’s instructions. miR-423-5p and PGM5-AS1 expression levels were analyzed by RT-qPCR.

### Luciferase reporter gene assay

Putative lncRNA-miRNA and miRNA-mRNA interactions were verified using luciferase reporter gene assays. The wild-type (WT) PGM5-AS1 (PGM5-AS1-WT) sequence containing miR-423-5p binding sites and its mutant sequences (PGM5-AS1-Mut) were chemically synthesized and inserted into pmirGLO-Report luciferase vectors (Synbio Tech). H460 and PC-9 cells were then co-transfected with the reporter vector and miR-423-5p mimic or mimic NC for 48 h. Luciferase activities were measured using the Dual-Luciferase Reporter Assay Kit (Promega, Madison, WI, USA) according to the manufacturer’s instructions.

### Western blot analysis

H460 and PC-9 cells were harvested and lysed in lysis buffer containing phosphatase inhibitors, proteinase inhibitor and PMSF (KeyGen Biotech). The protein concentration was determined using the BCA Protein Quantitation Assay Kit (KeyGen Biotech). Protein extracts were separated by SDS-polyacrylamide gel electrophoresis (SDS-PAGE), then transferred to PVDF membranes. Membranes were blocked with 5% skim milk, then incubated with a primary antibody against SLIT2 (1:500, ABclonal Technology, Wuhan, China). Following washing, the membrane was then incubated with peroxidase-conjugated affinipure goat anti-rabbit IgG. Protein bands were detected using the GelCapture version software (DNR Bio-Imaging Systems, Jerusalem, Israel). GAPDH (1:1000, Signalway Antibody, Maryland, USA) was used as the internal control for total protein input.

### Tumor xenograft model in nude mice

Female nude mice aged 4 weeks (Changsheng Biotech, Benxi, China) were used in this study and maintained under pathogen-free conditions. Stably-transfected H460 cells (5 × 10^6^) were subcutaneously injected into the nude mice. Tumors were measured every 7 days. Mice were humanely killed on day 28 and the tumors were removed. The tumor volume was calculated using the formula: volume = (A × B^2^)/2, where A is the longest diameter and B is the shortest diameter of the tumor. Tumors were weighed to obtain the tumor weight.

### Statistical analysis

Statistical analysis was performed using SPSS 20.0 software (IBM Corp, Armonk, NY, USA).All data are presented as mean value ± standard deviation. Student’s t-test was used to compare the variances between two groups, while one-way ANOVA was used to compare the means of three or more groups. All experiments were performed at least three times independently. A value of P < 0.05 was considered statistically significant.

## Results

### PGM5-AS1 is down-regulated in NSCLC and associated with patient prognosis

**Fig. 1:**
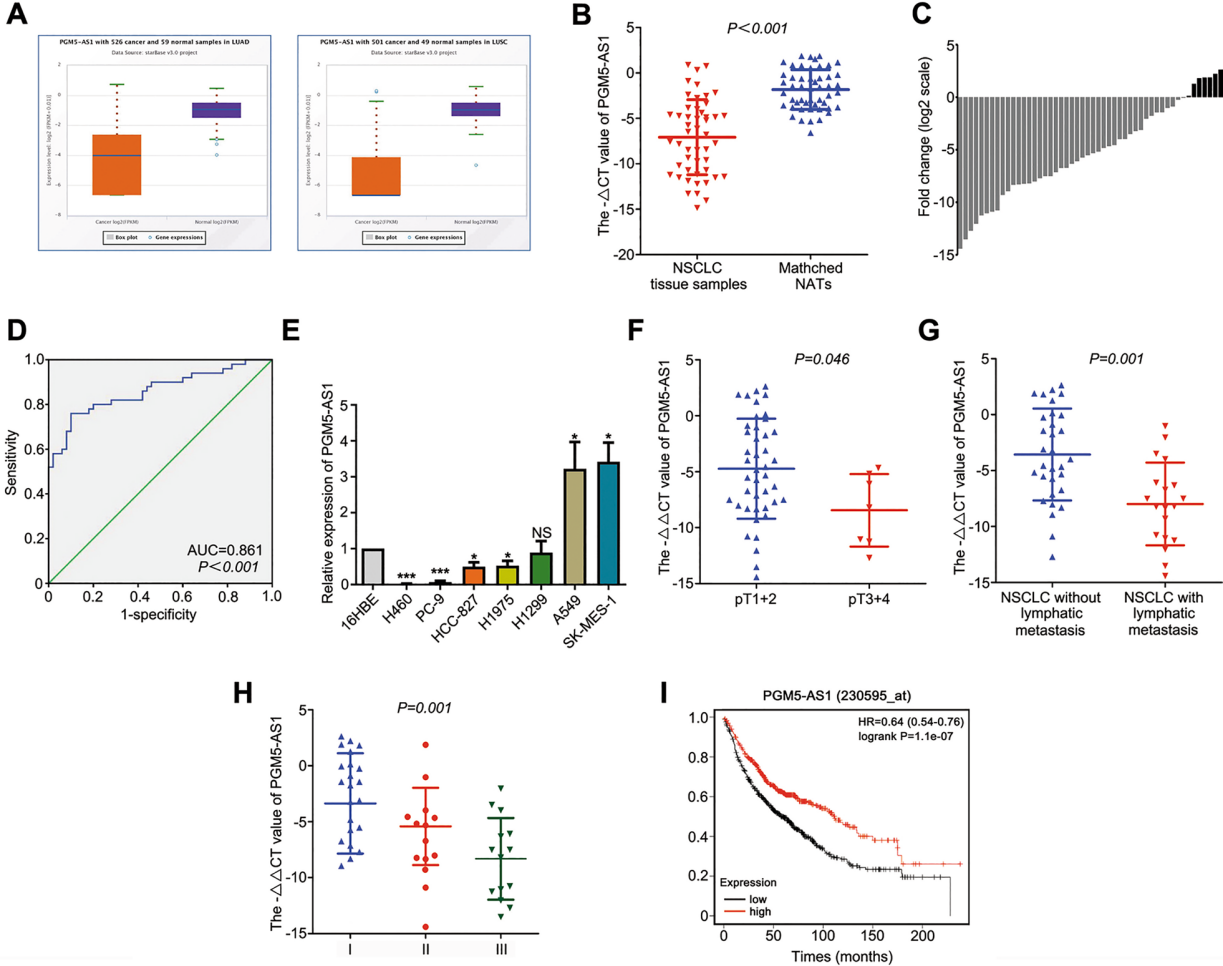
PGM5-AS1 is down-regulated in NSCLC and associated with patient prognosis. **A** PGM5-AS1 was down-regulated in LUAD and LUSC tissues compared with normal tissues, as determined by ENCORI analysis. **B** PGM5-AS1 expression levels in NSCLC and NATs were measured by RT-qPCR. **C** PGM5-AS1 was down-regulated in 43 of 50 patients with NSCLC. **D** ROC curve was constructed to estimate the diagnostic value in NSCLC. **E** PGM5-AS1 expression in normal 16HBE and NSCLC cell lines was measured by RT-qPCR. **F** Patients with lower PGM5-AS1 expression have larger tumor sizes. **G** Patients with lower PGM5-AS1 expression have positive lymphatic metastasis. **H** Patients with lower PGM5-AS1 expression have advanced TNM stage. **I** Patients with lower PGM5-AS1 expression have worse Prognosis

We analyzed the expression data of PGM5-AS1 in NSCLC from The Cancer Genome Atlas (TCGA) database using The Encyclopedia of RNA Interactomes (ENCORI) [21]. We found that PGM5-AS1 was significantly decreased in lung adenocarcinoma (LUAD) and lung squamous cell carcinoma (LUSC) compared with normal tissues with a fold-change of 0.24 and 0.09 in LUAD and LUSC, respectively (Fig. 1A). Next, we compared the relative expression levels of PGM5-AS1 in 50 paired NSCLC tissues and matched NATs. We found that PGM5-AS1 expression was significantly lower in NSCLC tissues compared with NATs (Fig. 1B) and that PGM5-AS1 was down-regulated in 43 of 50 NSCLC cohorts (Fig. 1C). Next, we constructed receiver operating characteristic (ROC) curves to determine whether PGM5-AS1 could be used as a candidate to distinguish cancer from non-cancer and obtained an area under the ROC curve (AUC) value of 0.861 (Fig. 1D). PGM5-AS1 expression levels were also found to be decreased in H460, PC-9, HCC-827 and H1975 cells (Fig. 1E). Next, we examined the correlation between PGM5-AS1 expression levels and the pathological characteristics of 50 NSCLC patients in our hospital. We found that PGM5-AS1 was significantly associated with tumor size, lymphatic metastasis, TNM stage and prognosis in NSCLC (Table 1). Patients with lower PGM5-AS1 expression levels were found to have larger tumor sizes (Fig. 1F), positive lymphatic metastasis (Fig. 1G), advanced TNM stage (Fig. 1H) and worse prognosis (Fig. 1I). Together, our findings indicated that PG5-AS1 is significantly down-regulated in NSCLC and that low expression of PGM5-AS1 is significantly associated with the worse overall survival of NSCLC patients.

**Table 1:** Correlation between PGM5-AS1 and clinicopathological characteristics in 50 NSCLC patients

	Number of patients (n = 50)	Expression level of PGM5-AS1^a^	*P*-value
Age			0.434
≤ 64	28	0.0233 (0.0020–0.2107)	
> 65	22	0.0232 (0.0044–0.6159)	
Sex			0.125
Male	35	0.0146 (0.0031–0.2423)	
Female	15	0.1073 (0.0055–0.9615)	
Grade of differentiation			0.311
Low	17	0.0187 (0.0034–1.0292)	
Middle	28	0.0262 (0.0035–0.3491)	
High	5	0.0005 (0.0003–0.0896)	
pT category			**0.046**
T1 + T2	43	0.0353 (0.0038–0.4914)	
T3 + T4	7	0.0034 (0.0004–0.0276)	
pN category			**0.001**
Negative	31	0.0633 (0.0094–0.9615)	
Positive	19	0.0034 (0.0005–0.0146)	
pTNM stage			**0.001**
I	22	0.3314 (0.0164–1.4241)	
II	14	0.0172 (0.0027–0.0476)	
III	14	0.0044 (0.0004–0.0268)	

^a^PGM5-AS1 median relative expression (25–75th percentile)

### PGM5-AS1 significantly inhibits cell proliferation and metastasis of NSCLC cell lines, and promotes cell apoptosis and G0/G1 cell cycle arrest

**Fig. 2:**
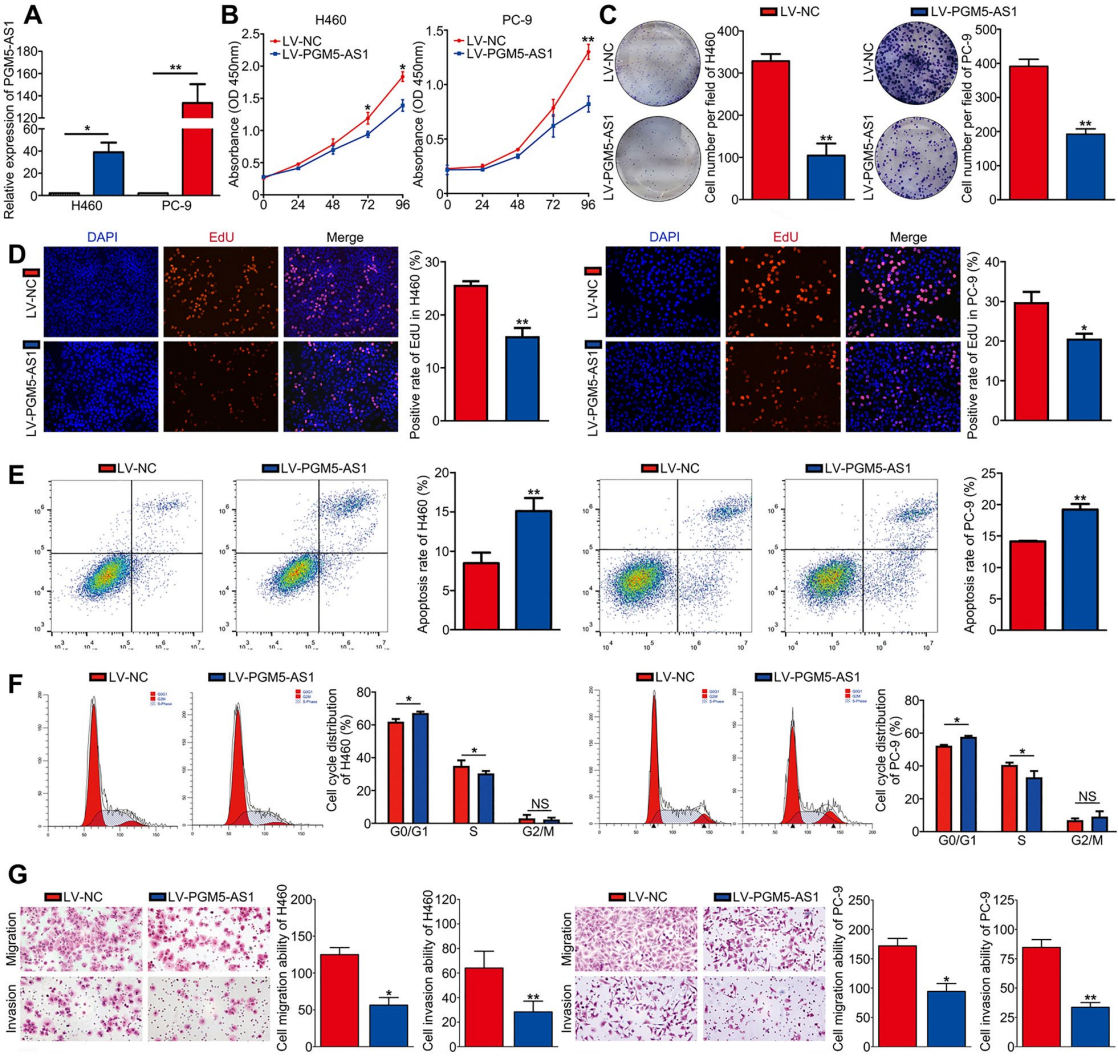
PGM5-AS1 inhibits cell proliferation and metastasis of NSCLC cell lines, and promotes cell apoptosis and G0/G1 cell cycle arrest. **A** H460 and PC-9 cell lines were transfected with lentivirus, and the transfection efficiency of these two groups was measured by qRT-PCR. **B** CCK-8 assay showed PGM5-AS1 inhibits NSCLC cell growth. **C** Colony formation assay showed PGM5-AS1 inhibits cell clonogenicity. **D** EdU assay showed PGM5-AS1 inhibits cell proliferation. **E** Cell apoptosis assay showed PGM5-AS1 increases the apoptosis rate. **F** Cell cycle assay showed PGM5-AS1 arrest cell cycle at G0/G1 phase. **G** Transwell assay showed PGM5-AS1 inhibits cell migration and invasion

Our preliminary studies indicated that the H460 and PC-9 cell lines displayed the lowest PGM5-AS1 expression levels of all the cell lines examined here. Thus, H460 and PC-9 cells were used in subsequent studies. Both cell lines were transduced with lentiviral vectors overexpressing PGM5-AS1. RT-PCR was used to measure the transduction efficiency and revealed that PGM5-AS1 expression was significantly increased following transduction with PGM5-AS1-overexpressing lentiviral vectors (Fig. 2A). Our CCK-8 and colony formation assays showed that overexpression of PGM5-AS1 significantly inhibited proliferation (Fig. 2B, C). Similarly, our EdU data indicated that the number of EdU-positive cells was lower in PGM5-AS1-overexpressing cells (Fig. 2D). Flow cytometric analysis of the apoptotic rate and cell cycle distribution revealed that overexpression of PGM5-AS1 led to a significant increase in apoptosis, as well as G0/G1 cell cycle arrest (Fig. 2E, F). Finally, we found that overexpression of PGM5-AS1 reduced the migratory and invasive abilities of H460 and PC-9 cells (Fig. 2G). Together, our results demonstrated that PGM5-AS1 inhibits the malignant behavior of NSCLC cells.

### PGM5-AS1 functions as a molecular sponge for miR-423-5p in NSCLC cells

**Fig. 3:**
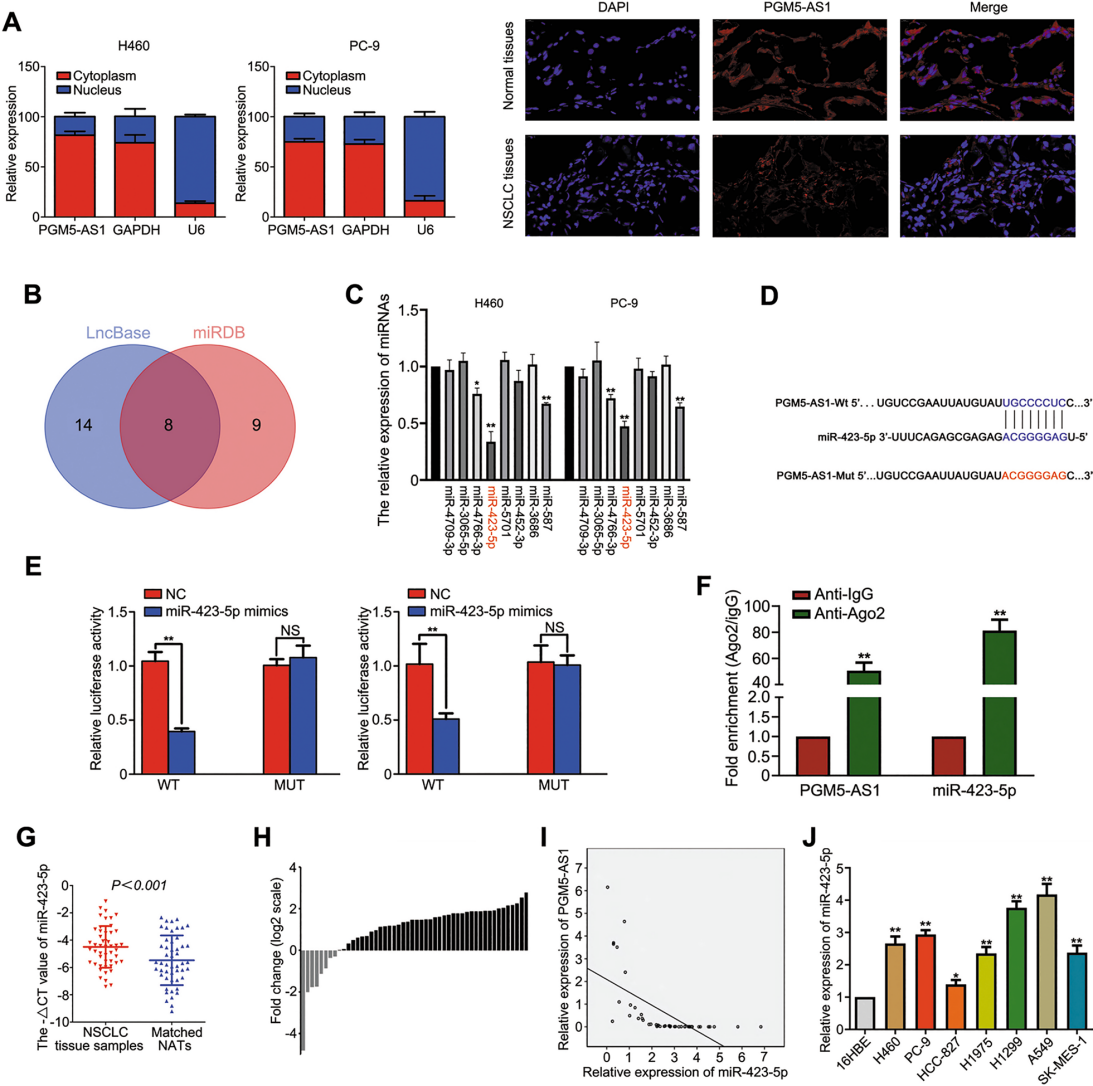
PGM5 AS1 functions as a molecular sponge for miR-423-5p in NSCLC cells. **A** Subcellular fractionation and FISH assay showed PGM5-AS1 was predominantly distributed in the cytoplasm. **B** Two circles represent the targeted miRNAs of PGM5-AS1 in DIANA Tools database and miRDB database. The middle part represents the intersection of miRNAs. **C** qRT-PCR measurement of the relative expression of the predicted miRNAs when PGM5-AS1 overexpression in NSCLC cells. **D** Predicted potential PGM5-AS1 binding sites and mutated nucleotides in the potential binding sequence of miR-423-5p in PGM5-AS1. **E** The binding of PGM5-AS1 to miR-423-5p was confirmed via dual-luciferase reporter gene assay. **F** PGM5-AS1 and miR-423-5p expressions were analyzed by RT-qPCR in H460 after AGO2-RIP analysis. **G** miR-423-5p expression levels in NSCLC and NATs were measured by RT-qPCR. **H** miR-423-5p is down-regulated in 4 of 50 patients with NSCLC. **I** The correlations between PGM5-AS1 and miR-423-5p in NSCLC tissues were analyzed using Pearson’s correlation. **J** miR-423-5p expression in normal 16HBE and NSCLC cell lines was measured by RT-qPCR

It is well-established that cancer-suppressive lncRNAs can interact with many cancer-promoting miRNAs and inhibit their functions [22]. Here, we performed subcellular fractionation to determine the cellular localization of PGM5-AS1. We found that PGM5-AS1 was predominantly distributed in the cytoplasm by subcellular fractionation and FISH assay, suggesting that PGM5-AS1 may play a regulatory role by acting as a miRNA sponge (Fig. 3A). Using the DIANA Tools database and miRDB (Fig. 3B), we identified eight potential miRNAs that potentially interacted with PGM5-AS1. miR-423-5p expression levels were found to be the most significantly reduced following overexpression of PGM5-AS1 (Fig. 3C). In addition, the miRDB database revealed a potential binding site between PGM5-AS1 and miR-423-5p (Fig. 3D). Thus, miR-423-5p was the focus of subsequent studies. Potential interactions between PGM5-AS1 and miR-423-5p were examined using the luciferase reporter assay. We found that the luciferase activity was decreased in cells co-transfected with miR-423-5p mimics and the PGM5-AS1-WT reporter, while no changes in luciferase activity were observed in cells co-transfected with the miR-423-5p mimics and the PGM5-AS1-mut reporter (Fig. 3E). AGO2-RIP analysis showed significantly higher enrichment of PGM5-AS1 and miR-423-5p in the AGO2 group than the IgG group (Fig. 3F). Taken together, the luciferase gene reporter assay and AGO2-RIP assay data demonstrated that PGM5-AS1 acts as a sponge for miR-423-5p. Furthermore, miR-423-5p was found to be up-regulated in NSCLC tissues and cell lines, indicating that miR-423-5p may have a regulatory role in NSCLC. PGM5-AS1 and miR-423-5p expression levels were found to be negatively correlated (Fig. 3G–I). Together, these results suggest that PGM5-AS1 functions as a molecular sponge of miR-423-5p in NSCLC.

### Overexpression of miR-423-5p reverses the suppressive effects of PGM5-AS1 on the malignant behavior of NSCLC cells

**Fig. 4:**
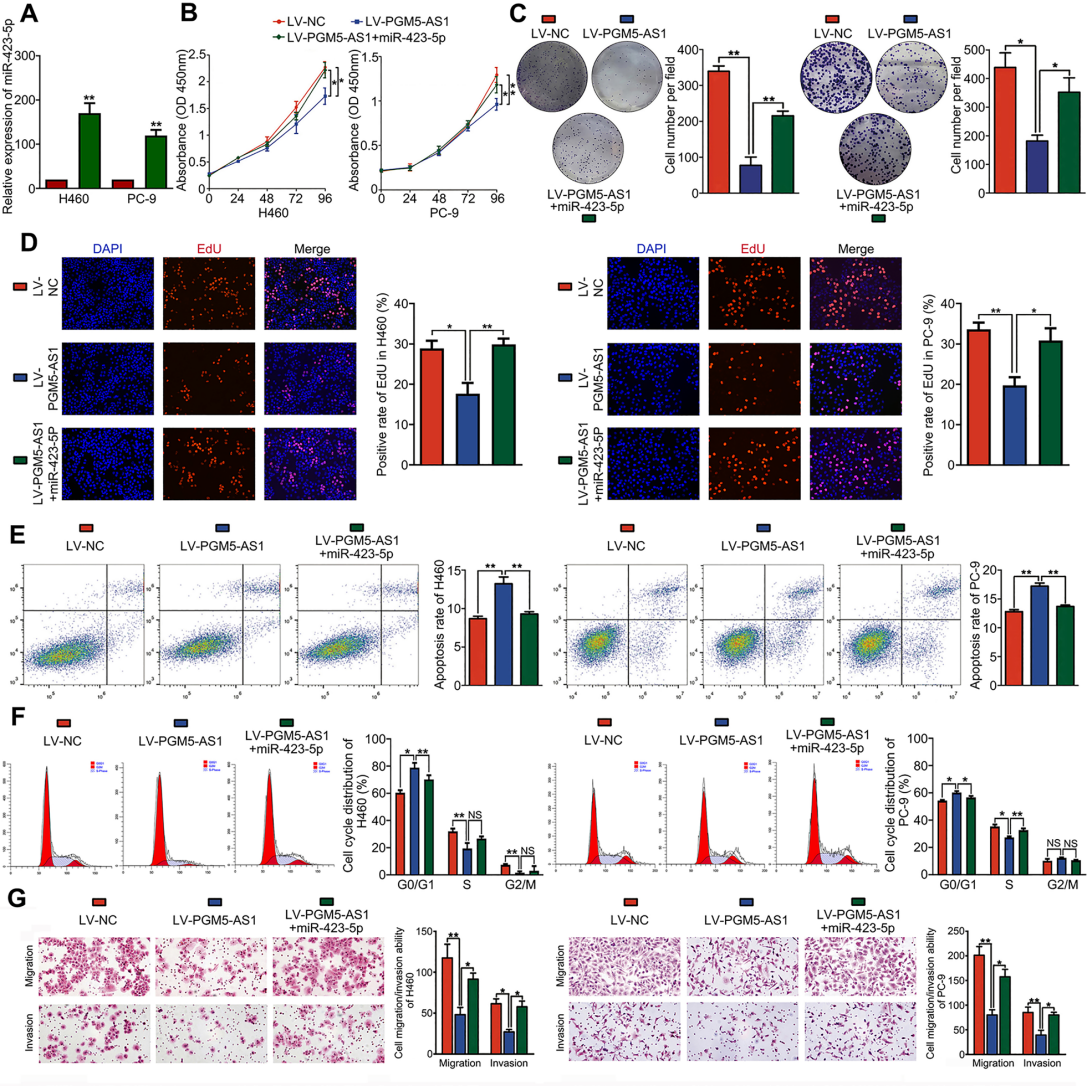
MiR-423-5p reverses the suppressive effects of PGM5-AS1 on the malignant behavior of NSCLC cells. **A** NSCLC cell lines were transfected with miR-423-5p mimics, and the transfection efficiency was measured by qRT-PCR. **B** CCK-8 assay showed miR-423-5p reversed the PGM5-AS1-induced suppressive effects on cell growth. **C** Colony formation assay showed miR-423-5p reversed the PGM5-AS1-induced suppressive effects on cell clonogenicity. **D** EdU assay showed miR-423-5p reversed the PGM5-AS1-induced suppressive effects on cell proliferation. **E** Cell apoptosis assay showed miR-423-5p weakened the PGM5-AS1-induced cell apoptosis rate increased. **F** Cell cycle assay showed miR-423-5p reversed the PGM5-AS1-induced G0/G1 arrest. **G** Transwell assay showed miR-423-5p reversed the PGM5-AS1-induced suppressive effects on cell migration and invasion

Next, we carried out rescue experiments to determine whether miR-423-5p is involved in regulating the effects of PGM5-AS1 in NSCLC. Using RT-PCR to measure the miRNA transfection efficiency, we showed that miR-423-5p expression levels were significantly increased following transfection with miR-423-5p mimics (Fig. 4A). Next, we showed that overexpression of miR-423-5p significantly reversed the PGM5-AS1-induced suppressive effects on proliferation using CCK-8, colony formation and EdU assays (Fig. 4B–D). Similarly, overexpression of miR-423-5p reversed the PGM5-AS1-induced increase in apoptosis and G0/G1 arrest (Fig. 4E, F). Finally, significantly higher cell migratory and invasive capacities were observed in cells overexpressing miR-423-5p and LV-PGM5-AS1 than in cells overexpressing LV-PGM5-AS1 alone (Fig. 4G). Overall, our findings indicate that miR-423-5p overexpression reverses the suppressive effects of PGM5-AS1 on malignant behavior.

### SLIT2 is a direct target gene of miR-423-5p in NSCLC

**Fig. 5:**
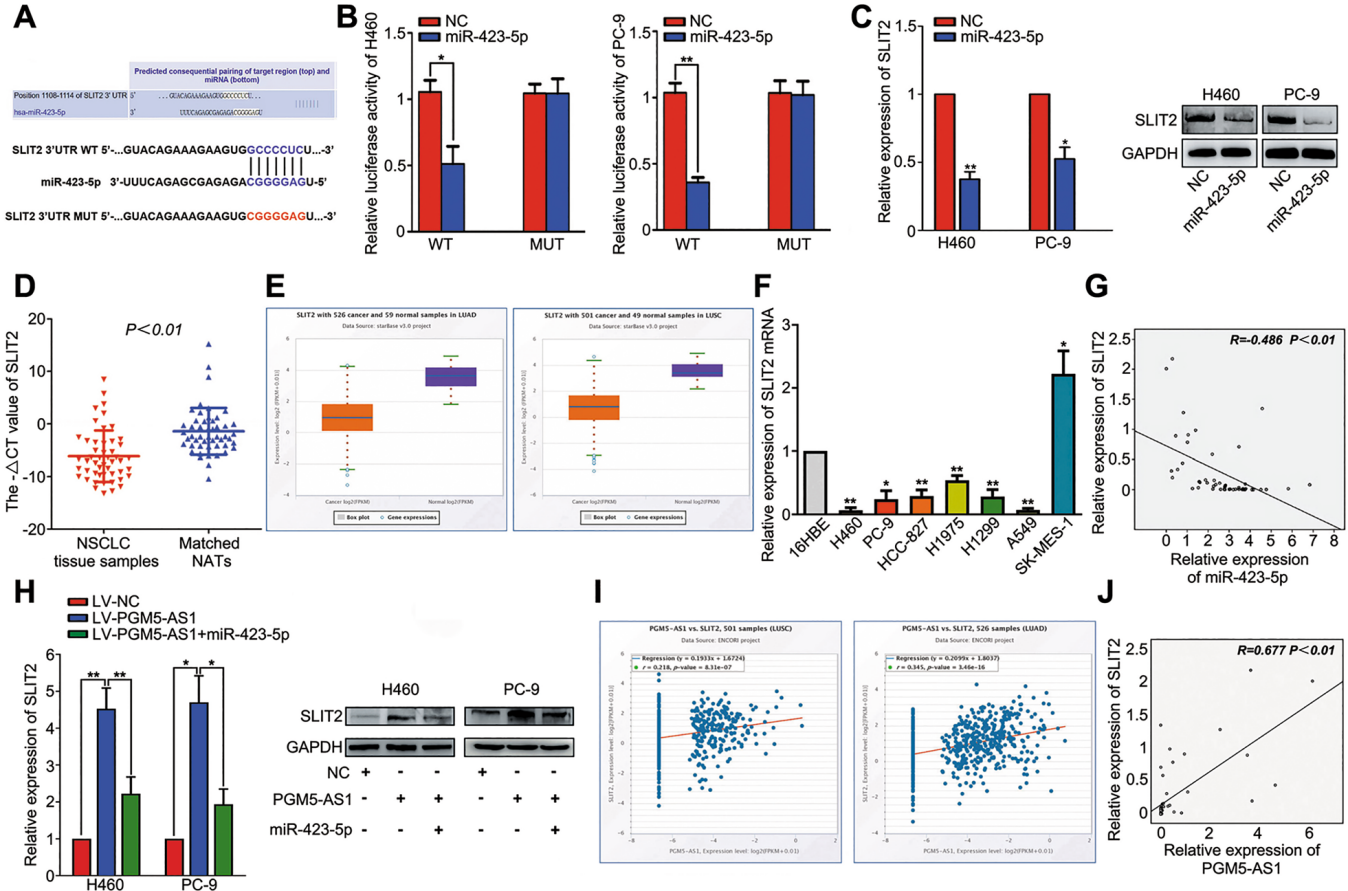
SLIT2 is a direct target gene of miR-423-5p in NSCLC. **A** Putative binding site between miR-423-5p and the 3ʹ-UTR of SLIT2 mRNA. **B** The binding site between miR-423-5p and SLIT2 3ʹ-UTR was verified by the luciferase assay. **C** qRT-PCR and Western blot measurement of the mRNA and protein levels of the SLIT2 when miR-423-5p overexpression in NSCLC cells. **D** SLIT2 expression levels in NSCLC and NATs were measured by RT-qPCR. **E** SLIT2 was down-regulated in LUAD and LUSC tissues compared with normal tissues, as determined by ENCORI analysis. **F** SLIT2 mRNA levels in 16HBE and NSCLC cell lines were measured by RT-qPCR. **G** The correlations between miR-423-5p and SLIT2 in NSCLC tissues were analyzed using Pearson’s correlation. **H** Overexpression of miR-423-5p reversed the PGM5-AS1-mediated increase in SLIT2 mRNA and Protein expression. **I** The correlations between PGM5-AS1 and SLIT2 in LUAD and LUSC were analyzed by ENCORI analysis. **J** The correlations between PGM5-AS1 and SLIT2 in NSCLC tissues were analyzed using Pearson’s correlation

miRNAs are known to play an important role in cancer progression by regulating the expression of their target genes. Here, we used TargetScan to predict the target genes of miR-423-5p, and identified SLIT2 as a target gene. SLIT2 has previously been reported to act as a tumor-suppressor in lung cancer, thyroid cancer, gastric cancer, acute promyelocytic leukemia and other types of cancer [19, 20, 23–25]. Thus, we hypothesized that SLIT2 is a target gene of miR-423-5p. The predicted interaction site is shown in Fig. 5A. Our luciferase reporter assay showed that the luciferase activity of cells co-transfected with WT-SLIT2 3′-UTR and miR-423-5p mimic was significantly lower than in cells co-transfected with WT-SLIT2 3′-UTR and NC, while no significant changes in luciferase activity were observed between cells co-transfected with miR-423-5p mimic and mutant SLIT2 3′-UTR and cells co-transfected with NC and mutant SLIT2 3′-UTR (Fig. 5B). These findings suggested that SLIT2 3′-UTR binds to miR-423-5p. We found that overexpression of miR-423-5p led to a significant reduction in SLIT2 mRNA and protein expression levels (Fig. 5C). In addition, we found that SLIT2 was significantly down-regulated in NSCLC tissues and cells, consistent with the ENCORI database data (Fig. 5D–F). Furthermore, the expression of miR-423-5p and SLIT2 was found to be negatively correlated (Fig. 5G). Next, we measured SLIT2 mRNA and protein expression levels in LV-NC-, LV-PGM5-AS1- and LV-PGM5-AS1 + miR-423-5p-transfected cells, and found that overexpression of miR-423-5p reversed the PGM5-AS1-mediated increase in SLIT2 expression (Fig. 5H). Moreover, the positive correlation between PGM5-AS1 and SLIT2 was verified in our cohort and the ENCORI database (Fig. 5I, J). Together, our findings identify SLIT2 as a potential target gene of miR-423-5p.

### Silencing SLIT2 reverses the cancer-suppressive effects induced by inhibition of miR-423-5p in NSCLC cells

**Fig. 6:**
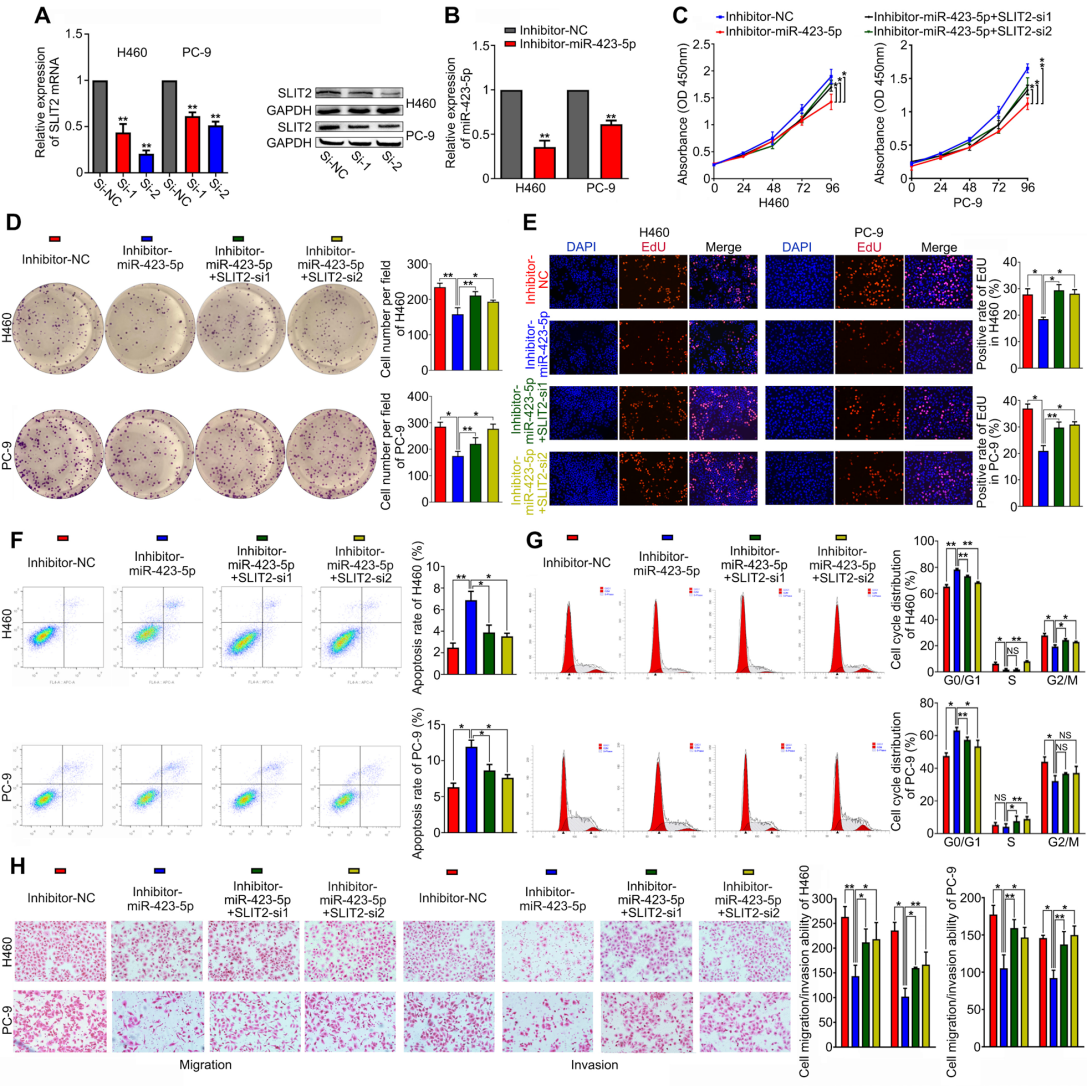
Silencing SLIT2 reverses the cancer-suppressive effects induced by inhibition of miR-423-5p. **A** NSCLC cell lines were transfected with SLIT2 siRNA, and the transfection efficiency was measured by qRT-PCR and Western blot. **B** NSCLC cell lines were transfected with miR-423-5p inhibitor, and the transfection efficiency was measured by qRT-PCR. **C–E** silencing miR-423-5p and SLIT2 simultaneously reversed the inhibitory effects of silencing miR-423-5p alone on cell proliferation by CCK-8, colony formation and EdU assays. **F–G** Cell apoptosis and cycle assay showed silencing miR-423-5p increased apoptotic rates and G0/G1-cell cycle arrested cells, while silencing both miR-423-5p and SLIT2 reversed the effects of miR-423-5p inhibition alone. **H** silencing miR-423-5p and SLIT2 simultaneously reversed the inhibitory effects of silencing miR-423-5p alone on cell migration and invasion

Next, we performed rescue experiments to determine whether SLIT2 was involved in mediating the biological effects of miR-423-5p. First, we showed that SLIT mRNA and protein expression levels were significantly reduced in H460 and PC-9 cells transfected with SLIT-si1 and SLIT-si2 (Fig. 6A). miR-423-5p expression levels were also significantly reduced following treatment with a miR-423-5p inhibitor (Fig. 6B). Proliferation was significantly reduced in miR-423-5p-inhibited cells compared to NC-treated cells as measured by CCK-8, colony formation and EdU assays, while silencing miR-423-5p and SLIT2 simultaneously was found to reverse the inhibitory effects of silencing miR-423-5p alone on cell proliferation (Fig. 6C–E). Moreover, silencing miR-423-5p led to significantly increased apoptotic rates and G0/G1-cell cycle arrested cells, while silencing both miR-423-5p and SLIT2 reversed the effects of miR-423-5p inhibition alone (Fig. 6F, G). Similarly, silencing both miR-423-5p and SLIT2 reversed the inhibitory effects induced by inhibition of miR-423-5p alone on cell migration and invasion (Fig. 6H). Together, our results show that knockdown of SLIT2 reverses the cancer-suppressive effects induced by inhibition of miR-423-5p.

### Overexpression of PGM5-AS1 suppresses tumor formation in vivo

**Fig. 7:**
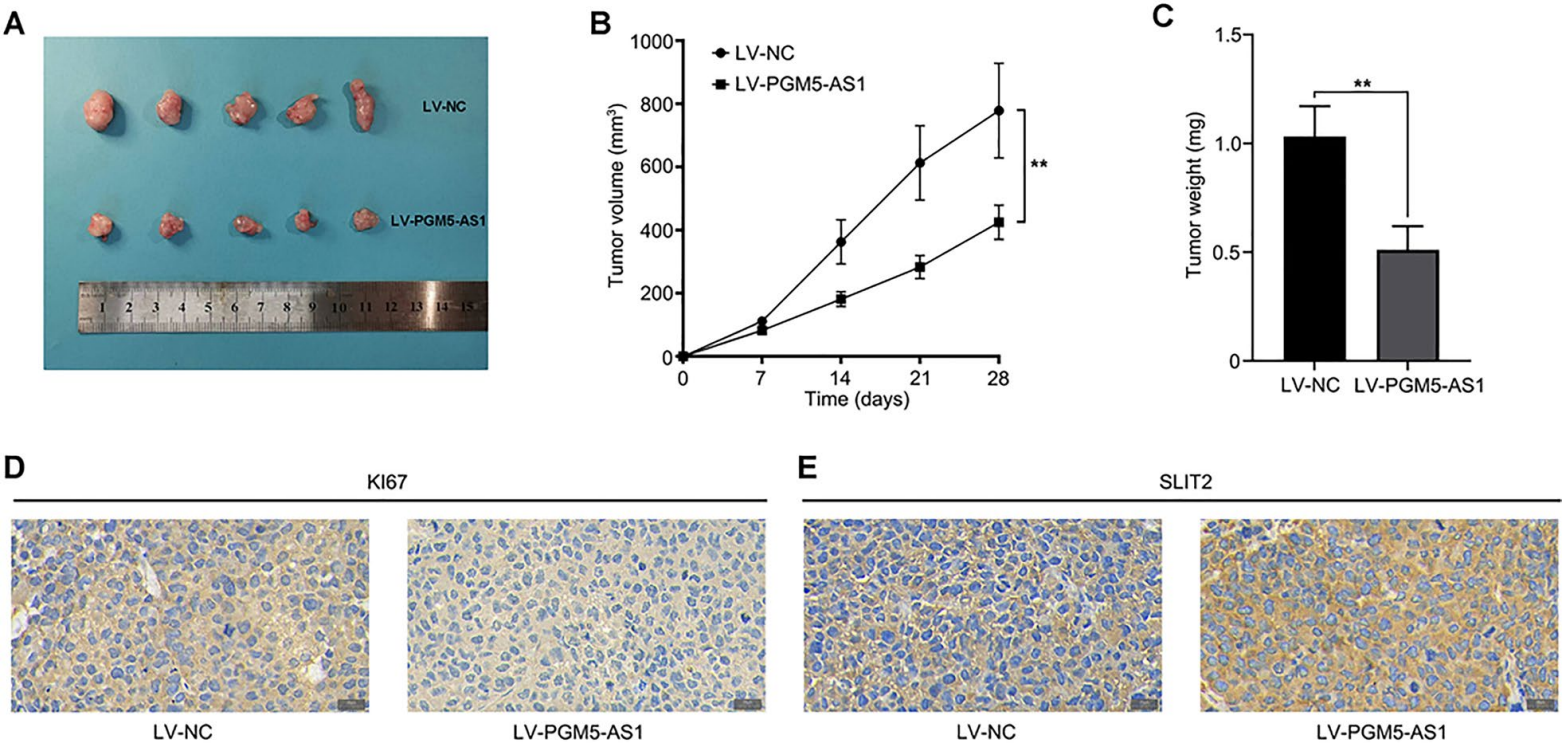
PGM5-AS1 can suppress the tumor formation in vivo. **A** PGM5-AS1 significantly inhibited the tumorigenicity in H460. **B**, **C** Tumor volume and weight at 28 d after injecting cells into mice. **D**, **E** Representative images of Ki-67 and SLIT2 staining in tumor

To determine whether PGM5-AS1 acts as a tumor suppressor in vivo, we established a tumor xenograft model by subcutaneously injecting stable-PGM5-AS1-overexpressing H460 cells and control cells into the dorsal side of nude mice. Consistent with our in vitro data, we found that overexpression of PGM5-AS1 significantly inhibited the tumorigenicity of H460 cells in vivo (Fig. 7A). In addition, the tumor volume and weight of PGM5-AS1-overexpressing tumors was significantly reduced compared with NC tumors (Fig. 7B, C). Moreover, PGM5-AS1-overexpressing tumors displayed lower Ki-67 levels than the control group (Fig. 7D). Finally, we found that SLIT2 expression levels were significantly higher in PGM5-AS1-overexpressing tumors than control tumors (Fig. 7E). These results suggested that the PGM5-AS1/miR-423-5p/SLIT2 axis is involved in regulating NSCLC progression. Specifically, our data revealed that PGM5-AS1 inhibits NSCLC tumorigenesis in vivo.

## Discussion

Lung cancer is among the most common respiratory disease in China [3, 26]. Smoking, genetic factors, occupational exposure and environmental factors are all risk factors for lung cancer [27, 28]. Since lung cancer diagnosis relies on computed tomography scans and the unsatisfactory sensitivity and specificity of traditional serum tumor biomarkers, most patients are usually diagnosed at an advanced stage. Surgery, chemotherapy and radiotherapy are still the main treatment options for NSCLC, even though only small improvements in advanced patient survival have been made [29]. Therefore, the identification of effective biomarkers and therapeutic targets is critical to improve clinical outcomes in NSCLC patients.

In recent years, accumulating evidence has shown that lncRNAs are closely associated with the initiation and progression of many human cancers. Previous studies have reported that PGM5-AS1 plays a key role in the development of ESCC, ccRCC and CRC [15–17]. However, the role of PGM5-AS1 in NSCLC has not been elucidated. In the current study, we found that PGM5-AS1 expression was significantly down-regulated in NSCLC tissues and cell lines. Furthermore, low PGM5-AS1 expression levels were associated with tumor size, positive lymph node metastasis and advanced TNM stages of NSCLC. Thus, PGM5-AS1 may play a tumor-suppressive role in NSCLC.

Numerous studies have demonstrated that lncRNAs are involved in the proliferation and invasiveness of cancer cells. For example, DSCAM-AS1 has been shown to modulate EPS8 expression to promote proliferation and inhibit apoptosis by acting as a ceRNA of miR-137 in breast cancer. Here, we found, using CCK-8 and Transwell assays, that overexpression of PGM5-AS1 inhibited cell proliferation and metastasis in NSCLC cell lines.

Recently, the construction of regulatory ceRNA networks or lncRNA-miRNA-mRNA axes has helped elucidate the underlying regulatory mechanisms of lncRNAs. In our study, we found using bioinformatics prediction tools that miR-423-5p was a potential target of PGM5-AS1. Previous studies have reported that patients expressing miR-423-5p might be at the highest risk for brain metastasis, and thus these patients are most likely to benefit from prophylactic cranial irradiation. miR-423-5p has been shown to promote brain metastasis in LUAD and inhibit MTSS1 expression [30]. Moreover, miR-423-5p has been found to aggravate LUAD by regulating CADM1 expression [31]. In our study, we found that miR-423-5p was expressed at significantly higher levels in NSCLC than NATs, and was negatively correlated with PGM5-AS1 levels. Moreover, our luciferase reporter gene assay data confirmed that miR-423-5p was a target of PGM5-AS1.

Bioinformatics screening predicted that SLIT2 was a downstream target gene of miR-423-5p. SLIT2 belongs to the slit family of secreted glycoproteins, which are ligands for the Robo family of immunoglobulin receptors [32]. SLIT2 has also been shown to act as a tumor suppressor in a range of cancers including NSCLC [19, 33, 34]. Although previous studies have shown that SLIT2 is downstream target gene of miR-218, miR-424 and other miRNAs [35, 36], the regulatory relationship between miR-423-5p and SLIT2 has not been reported to date. Here, our luciferase reporter gene assay confirmed that miR-423-5p interacts directly with the 3′-UTR of SLIT2, and that PGM5-AS1 regulates the miR-423-5P/SLIT2 axis.

In summary, our findings identify a role for the PGM5-AS1/miR-423-5p/SLIT2 axis in the progression of NSCLC. Thus, PGM5-AS1 may be a potential diagnostic biomarker and therapeutic strategy against NSCLC.

## Data Availability

The authors will supply the relevant data in response to reasonable requests.
